# Tyrosine kinase c-Abl regulates the survival of plasma cells

**DOI:** 10.1038/srep40133

**Published:** 2017-01-06

**Authors:** Yan-Feng Li, Shengli Xu, Yuhan Huang, Xijun Ou, Kong-Peng Lam

**Affiliations:** 1 Immunology Group, Bioprocessing Technology Institute, Agency for Science, Technology and Research, 138668 Singapore; 2 Department of Physiology, Yong Loo Lin School of Medicine, National University of Singapore, 117599 Singapore; 3 Department of Microbiology & Immunology, Yong Loo Lin School of Medicine, National University of Singapore, 117599 Singapore

## Abstract

Tyrosine kinase c-Abl plays an important role in early B cell development. Its deletion leads to reduced pro- and pre-B cell generation in mice. However, its function in B cell terminal differentiation remains unexplored. Here, we used c-Abl^f/f^ Aicda^cre/+^ mice, in which c-Abl is ablated only in antigen-activated B cells, to study the role of c-Abl in germinal center (GC) B and antibody-secreting plasma cell formation. Upon challenge with a model antigen, we found normal GC and memory B but reduced plasma cells and antigen-specific antibody response in the mutant mice. *In-vitro* studies revealed that plasma cells lacking c-Abl could be generated but did not accumulate in culture, indicative of survival defect. They also exhibited impaired STAT3 phosphorylation. The plasma cell defects could be rectified by introduction of Bim-deficiency or delivery of colivelin, a STAT3 activator, into c-Abl^f/f^ Aicda^cre/+^ mice. Hence, c-Abl signalling regulates the survival of plasma cells.

The tyrosine kinase c-Abl is a member of the Src family of non-receptor tyrosine kinases and is ubiquitously expressed across various tissues. It is encoded by the Abelson murine leukemia viral oncogene homolog 1, also known as *ABL1*, and is located on chromosome 9 in human and chromosome 2 in the mouse. C-Abl has been shown to regulate multiple facets of cell physiology, ranging from cell proliferation, survival and migration to intracellular processes such as cytoskeleton reorganisation and response to DNA damage^1^,^2^. Because of its critical functions in cell biology, abnormal c-Abl expression or activity could drive cell transformation. The t(9;22) chromosomal translocation in human leads to the formation of the *BCR-ABL* fusion gene that encodes a constitutively expressed and cytoplasmic-localized c-Abl kinase and has been shown to be critical for the development of chronic myelogenous leukemia (CML), acute lymphoid leukemia (ALL) and acute myelogenous leukemia (AML)^3^,^4^,^5^. In addition to the prototypic BCR-ABL1 fusion kinase, other chromosomal rearranged chimeric proteins involving c-Abl had been identified and in most instances, the mutations led to a constitutively active c-Abl that drives tumor progression^3^.

c-Abl has also been shown to play a role in normal B cell development. B-lymphopoiesis begins with the commitment of haematopoietic stem cells to the B cell-lineage and their differentiation to progenitor (pro) and precursor (pre) B cells when they attempt to rearrange and express immunoglobulin (Ig) heavy and light chain genes. The Abelson murine leukemia virus had long been used to transform pre-B cells to study these processes of gene rearrangements^6^,^7^. Once B cells assemble functional B-cell receptors, they progress to the immature B cell stage and transit from the bone marrow to the peripheral lymphoid organs. In secondary lymphoid tissues, B cells encounter specific antigens and are activated with or without T cell-help to differentiate into antibody-secreting plasma cells (PC)^8^,^9^. In a T cell-dependent immune response, B cells received help from T cells and participate in a germinal center (GC) reaction where they undergo somatic hypermutation and Ig heavy chain class-switching to generate high affinity antibodies with different effector functions^10^. The GC reactions also give rise to memory B cells and long-lived PCs that home to the bone marrow^11^,^12^,^13^,^14^.

The expression of c-Abl was found to be uniform throughout B cell development but its activity peaked at the pre-B cell stage^15^,^16^. A role for c-Abl in early B cell development was evidenced by the study of germline c-Abl gene knockout in mice. In these mutants, B cell differentiation was impaired at the pro- and pre-B cell stages and peripheral B cell population was drastically reduced^15^,^16^,^17^,^18^. Transgenic expression of Ig heavy and light chains failed to rescue the B cell defects in these mice. Although it was clear from these studies that c-Abl signalling was critically important for early B cell development, it was unknown however, if c-Abl was needed for the terminal differentiation of B cells into GC and memory B cells as well as PCs. C-Abl is phosphorylated downstream of various growth factor and cytokine receptors and integrins and could be important for some aspects of B cell terminal differentiation or survival or functions of these cells^1^,^2^,^19^.

The c-Abl germline knockout mice were unsuitable for use in studying the role of c-Abl tyrosine kinase in late B cell differentiation and function as the mutant mice already manifested skewed early B cell development. Hence, to study the role of c-Abl in the later stages of B cell differentiation, we have generated c-Abl^f/f^Aicda^cre/+^ mice in which c-Abl is deleted only in activated B cells when they bind specific antigens. We showed that c-Abl is dispensable for the formation of GC B and memory B cells but it is critically important for the survival of PCs.

## Results

### Generation of c-Abl^f/f^Aicda^cre/+^ mice

**Figure 1:**
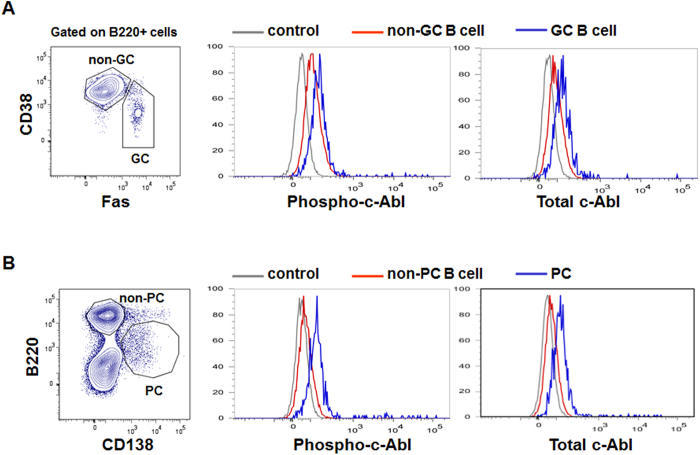
Flow cytometry analyses of total and phosphorylated c-Abl levels in GC B and plasma cells. Wildtype C57BL/6 mice were immunized with NP_38_-CGG and examined 10 days later for intracellular levels of total and phosphorylated c-Abl (phopho-c-Abl) levels in gated (**A**) Fas^+^ CD38^−^B220^+^ GC (blue histogram) and Fas^−^CD38^+^ B220^+^ non-GC B cells (red histogram) and (**B**) B220^low^CD138^+^ PC (blue histogram) and B220^high^CD138^−^ non-PC cells (red histogram). Staining with rabbit IgG antibody (gray histogram) was included as negative control. Data shown are representative of 3 independent experiments.

It was shown previously that c-Abl was expressed uniformly throughout B cell development and its activity peaked at the pro-B cell stage^15^. Correspondingly, c-Abl-deficient mice had defects in early B cell maturation resulting in reduced population of B cells^15^,^17^,^18^. However, the role of c-Abl in late stage B cell differentiation has not been explored. To begin to address this issue, we immunized C57Bl/6 mice with a T cell-dependent antigen, NP_38_-CGG in alum and used the same anti-c-Abl and anti-phospho-c-Abl antibodies that were used previously^15^ to examine c-Abl expression and activity in GC B and plasma cells. As shown in Fig. 1A, B220^+^Fas^+^CD38^−^ GC B cells expressed higher level of c-Abl protein and activity compared to non-GC B cells. Likewise, B220^+^CD138^low^ plasma cells (PC) also expressed higher levels of c-Abl protein and activity compared to non-PC (Fig. 1B). These data suggest that c-Abl might play a role in the terminal stages of B cell differentiation.

We next generated c-Abl^f/f^Aicda^cre/+^ mice in which the loxP-flanked *c-Abl* gene loci remain intact in naïve B cells but are deleted when B cells bind antigens and become activated to express the AID promoter-driven Cre recombinase. This strategy allowed us to directly assess the role of c-Abl in late stage B cell differentiation following activation. It also allowed us to bypass the B cell defects seen in c-Abl total knockout mice^15^,^16^,^17^,^18^.

**Figure 2:**
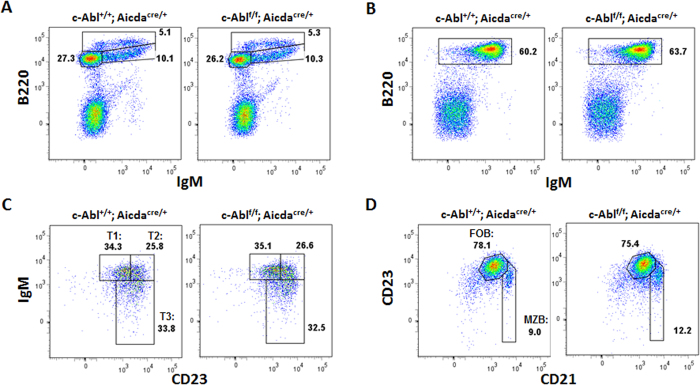
Examination of B cell populations in control and c-Abl^f/f^ Aicda^cre/+^ mice. B cell populations in the (**A**) bone marrow and (B-D) spleens of c-Abl^+/+^ Aicda^cre/+^ (control) and c-Abl^f/f^ Aicda^cre/+^ mice were determined using Flow cytometry. (**A**) Bone marrow cells were interrogated for expression of B220 and IgM. Numbers adjacent to gated areas indicate percent of total bone marrow cells. (**B**) Splenocytes were gated for B220^+^ IgM^+^ B cells and numbers shown indicate percent of total splenocytes. (**C**) CD19^+^ splenocytes were further interrogated for CD23 and IgM expression to define transitional T1 (CD23^−^IgM^+^), T2 (CD23^+^IgM^+^) and T3 (CD23^+^IgM^−^) subsets. (**D**) Gated splenic CD19^+^ cells were interrogated for CD23 and CD21 expression level. Follicular B (CD23^+^CD21^+^) and marginal zone B cells (CD23^−^CD21^++^) were determined. Numbers shown are percent of CD19^+^ B cells. Data shown are representative of 3 independent experiments.

Before we examine the function of c-Abl in B cell activation, we first ascertained that B cell development was indeed unperturbed in c-Abl^f/f^Aicda^cre/+^ mice. As shown in Fig. 2A, c-Abl^f/f^Aicda^cre/+^ mice have intact B-lymphopoiesis in the bone marrow with normal populations of B220^low^IgM^−^ pro/pre-B, B220^low^IgM^+^ immature B and B220^+^IgM^+^ circulating mature B cells. Peripheral IgM^+^ B cell population was also found to be comparable in the spleens of c-Abl^+/+^Aicda^cre/+^ (control) and c-Abl^f/f^Aicda^cre/+^ mice (Fig. 2B) with equivalent representation of transitional B cells at B220^+^CD23^−^IgM^+^T1, B220^+^CD23^+^IgM^+^T2 and B220^+^ CD23^+^ IgM^−^ T3 stages (Fig. 2C). In addition, CD23^+^CD21^+^ follicular and CD23^−^CD21^++^ marginal zone B cell subsets were also found to be intact in the spleens of c-Ablf/fAicda^cre/+^ mice (Fig. 2D). Taken together, the data indicated that c-Abl^f/f^Aicda^cre/+^ mice have normal B cell development and B cell subsets and therefore could be used as a good mouse model to study the role of c-Abl in B-cell activation and terminal differentiation.

### c-Abl deficiency does not affect GC and memory B cell generation

**Figure 3:**
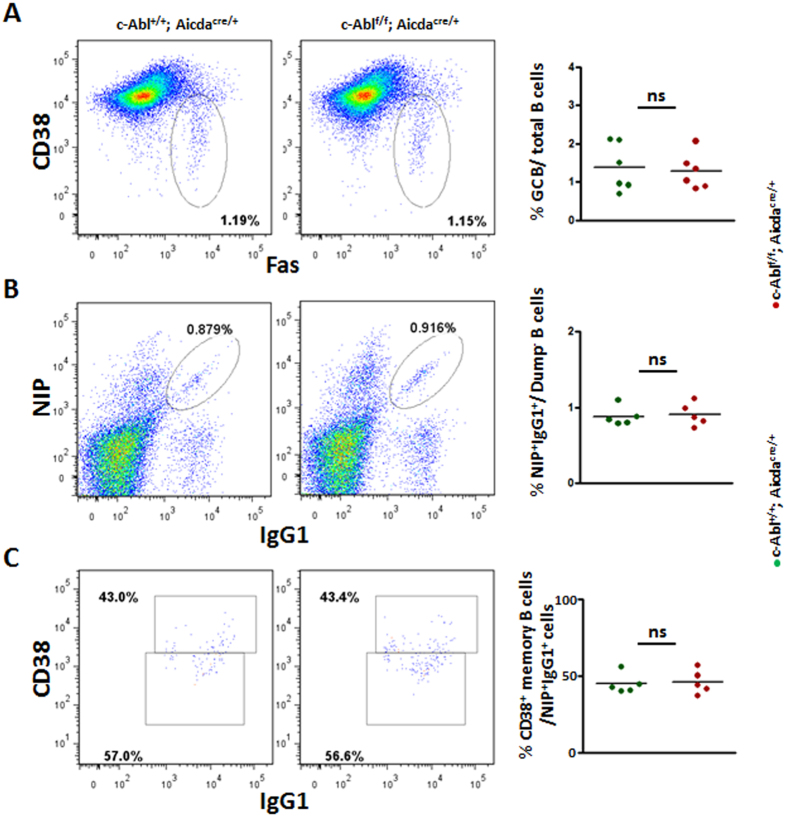
Analysis of GC and memory B cells in immunized control and c-Abl^f/f^ Aicda^cre/+^ mice. Control and c-Abl^f/f^ Aicda^cre/+^ mice were challenged with NP_38_-CGG and analysed 10 days after immunization. (**A**) Flow cytometric and statistical analyses of B220^+^Fas^+^CD38^−^ GC B cells in the spleens of immunized mice. Numbers shown are percent of B220^+^ cells. (**B**) Flow cytometric analysis and quantification of NP-specific NIP^+^IgG1^+^ B cells in the spleens of immunized mice. Numbers shown are percent of NIP^+^IgG1^+^ B cells outside of the B220^+^Dump^−^ gate. (**C**) Flow cytometric analysis of NIP^+^IgG1^+^ cells to further determine CD38^+^ memory B and CD38^−^ GC B cells. Numbers indicate percent of B220^+^Dump^−^NIP^+^IgG1^+^ spleen cells analysed. Quantification of antigen specific CD38^+^IgG1^+^NIP^+^ memory B cells is shown. Each dot in the 3 graphs represent one animal analysed; ns, non-significant.

The increased c-Abl phosphorylation in GC B cells (Fig. 1A) prompted us to examine GC B cell differentiation in c-Abl^f/f^Aicda^cre/+^ mice. We immunized control and c-Abl^f/f^Aicda^cre/+^ mice with a T cell-dependent antigen, NP_38_-CGG in alum, and examined GC reaction in the spleens 10 days after. First of all, we examined if c-Abl was deleted in GC B (B220^+^CD38^−^Fas^+^) cells of c-Abl^f/f^Aicda^cre/+^ mice. Expression of intracellular c-Abl was analysed by flow cytometry and we found that c-Abl expression level was indeed reduced in GC B cells of c-Abl^f/f^Aicda^cre/+^ mice compared to that of control mice (Supplementary Fig. 1A). We also examined the efficiency of AID-Cre-mediated deletion of c-Abl by qRT-PCR analysis using RNA extracted from FACS-sorted GC B cells from control and c-Abl^f/f^Aicda^cre/+^ mice. The qRT-PCR analyses further confirmed successful deletion of c-Abl in GC B cells sorted from c-Abl^f/f^Aicda^cre/+^ mice as compared to control mice (Supplementary Fig. 1B). Interestingly, we found that the B220^+^CD38^−^Fas^+^ GC B cell fractions were not perturbed in the spleens of c-Abl^f/f^Aicda^cre/+^ mice (Fig. 3A). Further examination of antigen-specific, class-switched NIP^+^IgG1^+^ B cells confirmed that the antigen-specific IgG1 GC (NIP^+^IgG1^+^CD38^−^) and memory B (NIP^+^IgG1^+^CD38^+^) cell populations were largely comparable between c-Abl^f/f^Aicda^cre/+^ and control mice (Fig. 3B and C). Taken together, our data indicated that c-Abl was not required for GC B and memory B cell formation.

### Impaired humoral immune response and plasma cell generation in c-Abl^f/f^Aicda^cre/+^ mice

**Figure 4:**
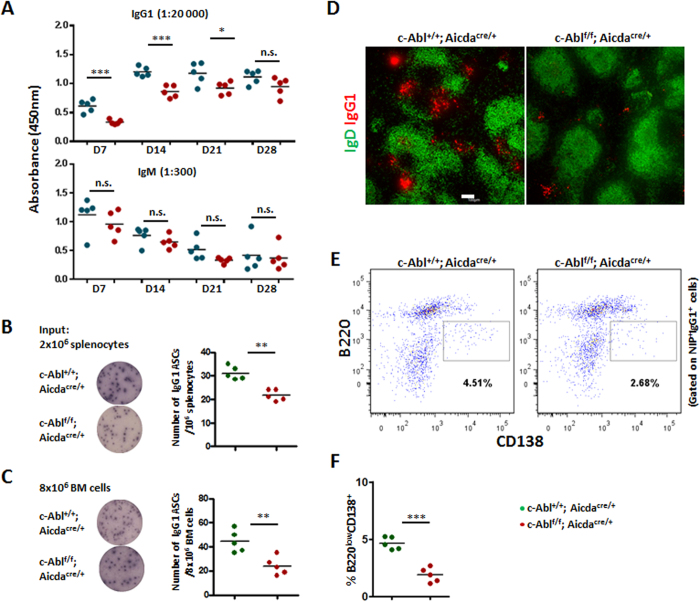
Impaired antibody response and reduced PC in c-Abl^f/f^ Aicda^cre/+^ mice. (**A**) Control and c-Abl^f/f^ Aicda^cre/+^ mice were challenged with NP_38_-CGG and their sera antigen-specific IgG1 and IgM antibody levels at different time-points after immunization were determined via ELISA. Sera were diluted 20,000 and 300 times respectively, to measure NP-specific IgG1 and IgM antibodies. (**B** and **C**) ELISPOT analyses and quantification of antigen-binding IgG1 antibody-secreting cells (ASC) in the spleens (**B**) and bone marrow (**C**) of control and c-Abl^f/f^ Aicda^cre/+^ mice 14 days after NP_38_-CGG immunization. (**D**) Confocal microscopy of spleen sections of NP_38_-CGG-challenged control and c-Abl^f/f^ Aicda^cre/+^ mice at day 14 of immunization to visualize IgG1 PC (red, anti-IgG1) and B cell follicles (green, anti-IgD); white bar, 100 μm. Images shown are representative of three spleens analysed per group. (**E**) Flow cytometry analysis of NIP^+^IgG1^+^ spleen cells to further depict B220^low^CD138^+^ plasma cells in day 14 immunized mice. (**F**) Quantification of antigen-specific B220^low^CD138^+^ PC in the spleens of day 14 immunized control and c-Abl^f/f^ Aicda^cre/+^ mice. Each dot represents one mouse analysed. *p < 0.05; **p < 0.01; ***p < 0.001.

We also examined if c-Abl^f/f^Aicda^cre/+^ mice could mount an effective antibody response upon challenge with antigen. We assayed for NP-specific IgG1 and IgM antibodies in the mice immunized with NP_38_-CGG at various time points post-immunization. Interestingly, as shown in Fig. 4A, c-Abl^f/f^Aicda^cre/+^ mice exhibited defective amount of NP-specific IgG1 antibodies from day 7 onwards till day 21 after primary immunization (Fig. 4A, upper panel). However, the titers of NP-specific IgM antibodies were comparable between c-Abl^f/f^Aicda^cre/+^ and control mice (Fig. 4A, lower panel), and this observation could be due to the fact that AID is only induced in B cells undergoing class-switching and therefore floxed-c-Abl alleles are not deleted in IgM PCs (Supplementary Fig. 2). Taken together, the ELISA data suggested that c-Abl function is required for class-switched IgG1 antibody production during T-cell–dependent humoral immune response.

Since NP-specific NIP^+^IgG1^+^ GC B cells were present in immunized c-Abl^f/f^Aicda^cre/+^ mice (Fig. 3B) and yet they exhibited reduced NP-specific IgG1 antibodies in the sera (Fig. 4A), we wondered if antibody-secreting PC would be generated normally in these mice. Hence, we interrogated NP-specific IgG1 PCs in immunized c-Abl^f/f^Aicda^cre/+^ mice via ELISPOT. Consistent with the ELISA data, we found significantly reduced numbers of NP-specific IgG1 PC in the spleen (Fig. 4B) and bone marrow (Fig. 4C) of c-Abl^f/f^ Aicda^cre/+^ mice compared with control mice at day 14 post-immunization. This finding was further confirmed by confocal microscopic study when we stained for intracellular IgG1-expressing cells in spleen tissue sections. Substantial clusters of IgG1-expressing PC were seen in the immunized control mice but these cells were drastically diminished in c-Abl^f/f^ Aicda^cre/+^ mice (Fig. 4D). We also managed to detect a distinct population of NIP^+^IgG1^+^B220^low^CD138^+^ PC in immunized control mice (Fig. 4E), but this population was found to be greatly reduced in c-Abl^f/f^Aicda^cre/+^ mice (Fig. 4F). These data together suggest that c-Abl might play an important role in plasma cell biology and its absence leads to impaired antibody production during humoral immune response.

### PC lacking c-Abl could be generated but did not accumulate *in vitro*

**Figure 5:**
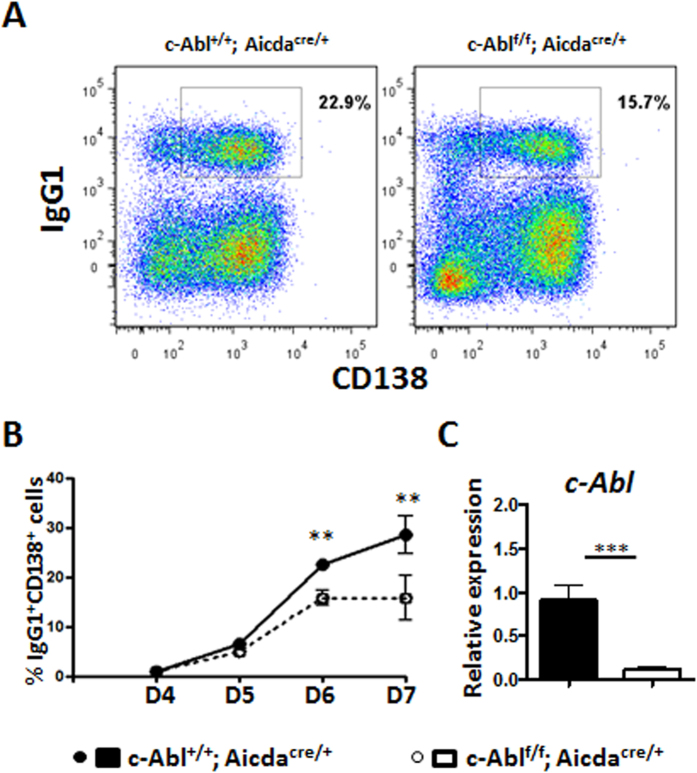
PC lacking c-Abl can be generated *in-vitro* but did not accumulate in culture. Naïve B cells were purified from control and c-Abl^f/f^ Aicda^cre/+^ mice and stimulated with soluble CD40 ligand (100 ng/ml), IL-4 (10 ng/ml) and IL-5 (5 ng/ml). At day 3 of culture, IL-5 (5 ng/ml) and IL-21 (10 ng/ml) were added and cells were analyzed from day 4 to 7. (**A**) Intracellular staining and FACS analyses to identify CD138^+^IgG1^+^ plasma cells at day 6 of culture. (**B**) Quantification of CD138^+^IgG1^+^ PC in cultures from day 4 to 7. Group of five experiments were analyzed, and data were expressed as mean ± s.e.m. (**C**) qRT-PCR analysis showing the deletion of c-Abl alleles in the *in-vitro* culture.

The lack of PC in immunized c-Abl^f/f^ Aicda^cre/+^ mice could be due to defective generation or survival of these cells. To address this issue, we attempted to differentiate c-Abl-deficient PC *in-vitro*. Naïve B cells were purified from control and c-Abl^f/f^ Aicda^cre/+^ mice and stimulated for 3 days with IL-4, IL-5, and soluble CD40 ligand which mimic T cell-help. Thereafter, additional IL-5 and IL-21 were added to the culture at day 4 to induce PC differentiation^20^,^21^. After 6 days of culture, we were able to induce a significant population of IgG1^+^CD138^+^ PCs in the control culture (Fig. 5A). We were also able to detect a distinct population of PCs in the c-Abl^f/f^Aicda^cre/+^ culture. We determined the fraction of PCs in control and c-Abl^f/f^Aicda^cre/+^ cultures from day 4 onwards and showed that mutant PCs could be generated but they did not accumulate in culture (Fig. 5B). Again, we performed qRT-PCR to confirm that the c-Abl was indeed deleted in the cells cultured from c-Abl^f/f^Aicda^cre/+^ mice (Fig. 5C). These data suggested that c-Abl-deficient PCs likely have a survival defect.

### c-Abl-deficiency affects STAT3 activation in PC

**Figure 6:**
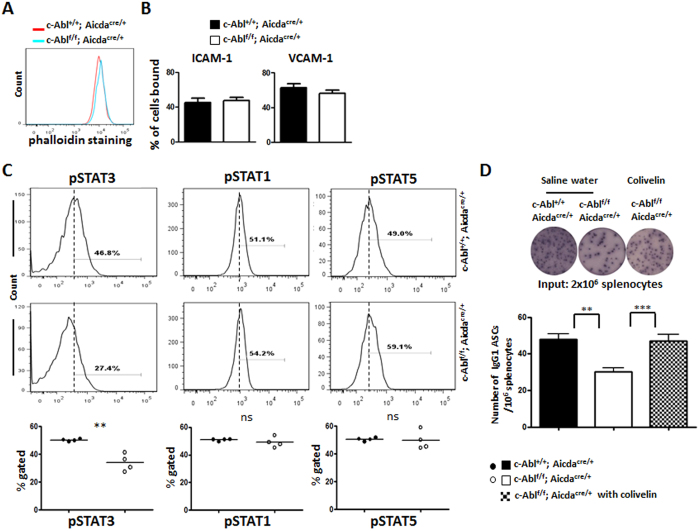
Defective STAT3 signalling in c-Abl-deficient PC. Analyses of various signaling pathways in *in vitro* generated PC harvested at day 6 of culture. (**A**) Intracellularly-stained IgG1^+^CD138^+^ PC were examined for their actin structure by co-staining with phalloidin in FACS analysis in control (red histogram) and c-Abl^f/f^ Aicda^cre/+^ mice (blue histogram). (**B**) Flow cytometry analysis of soluble VCAM-1-Fc (20 μg/ml) and ICAM-1-Fc (20 μg/ml) binding by *in-vitro* differentiated cells from control (black columns) and c-Abl^f/f^ Aicda^cre/+^ (white columns) mice. (**C**) IgG1^+^CD138^+^ PC generated from control and c-Abl^f/f^ Aicda^cre/+^ B cell cultures were examined for levels of STAT1, STAT3 and STAT5 phosphorylation. Quantification of the percentage of phosphorylated STAT1, STAT3 and STAT5 in control (black circles) and c-Abl^f/f^ Aicda^cre/+^ (white circles) PC. (**D**) ELISPOT and statistical analyses of NP-specific PCs in the spleens of control, c-Abl^f/f^ Aicda^cre/+^, and c-Abl^f/f^ Aicda^cre/+^ mice treated with the STAT3-activator Colivelin. 5 days after NP_38_-CGG challenge, c-Abl^+/+^ Aicda^cre/+^ and c-Abl^f/f^ Aicda^cre/+^ mice were injected with the vehicle control saline buffer and/or STAT3 activator Colivelin (dosage at 1.5 μg/g body weight). Each circle represents one set of experiment performed.

The tyrosine kinase c-Abl is known to regulate various aspects of cellular biology ranging from actin reorganization, autophagy formation to integrin signalling^2^,^22^,^23^,^24^. Defects in any of these functions could possibly affect PC survival and persistence. To gain more insights into the molecular basis of the PC defect in c-Abl^f/f^Aicda^cre/+^ mice, we studied in detail the PC generated in the *in vitro* culture. First, we examined actin structures in control and mutant PCs by staining them with phalloidin and they appeared to be comparable (Fig. 6A).

We also examined integrin function in c-Abl-deficient PCs as we and others had shown that α4β1 and αLβ2 are particularly important for PC homing and persistence in the bone marrow^13^,^14^. However, the function of both integrins α4β1 and αLβ2, as reflected by their abilities to bind their respective ligands, were intact in PCs lacking c-Abl (Fig. 6B).

Finally, we studied STAT3 signalling in c-Abl-deficient PCs as c-Abl and its chimeric oncoproteins had been shown to regulate the activation of STAT3 in cancer cells^26^,^27^,^28^ and separately, STAT3 has been shown to regulate PCs survival in several recent studies^29^,^30^,^31^. Analyses of intracellular STAT3 activation in the *in vitro* differentiated PCs revealed that the phosphorylation of STAT3 was compromised in mutant compared to control PCs (Fig. 6C). The defect was specific for STAT3 activation as the phosphorylation of STAT1 and STAT5 were comparable between the control and mutant PCs.

The data so far suggested that defective c-Abl-dependent STAT3 activation underlies the impaired PC survival in c-Abl^f/f^Aicda^cre/+^ mice. Previous studies showed that Colivelin, a 26-amino-acid peptide, could interact and activate STAT3, thus bypassing upstream signalling defect that can cause impaired STAT3 activation^32^,^33^. We next examined if the delivery of Colivelin could rectify the PC defect in the immunized c-Abl^f/f^ Aicda^cre/+^ mice. Mice were first challenged with NP_38_-CGG at day 0 and given the vehicle control saline buffer or Colivelin at day 5, when PCs start to accumulate in the spleen, and sacrificed at day 10 post-immunization (5 days post-treatment) for PC analysis via ELISPOT. In agreement with previous data, the number of PC in the spleens of immunized c-Abl^f/f^Aicda^cre/+^ mice given control treatment was very much reduced compared with similarly treated c-Abl^+/+^Aicda^cre/+^ mice (Fig. 6D). Interestingly, the number of PCs was significantly increased in the spleens of c-Abl^f/f^Aicda^cre/+^ mice given Colivelin compared to that of c-Abl^f/f^Aicda^cre/+^ mice given saline. Hence, these results suggested that defective STAT3 signalling was indeed responsible for the PC defect in c-Abl^f/f^Aicda^cre/+^ mice. Taken together, these data indicate that c-Abl signals via STAT3 to regulate PC survival.

### Bim-deficiency rescues PC defect in c-Abl^f/f^ Aicda^cre/+^ mice

**Figure 7:**
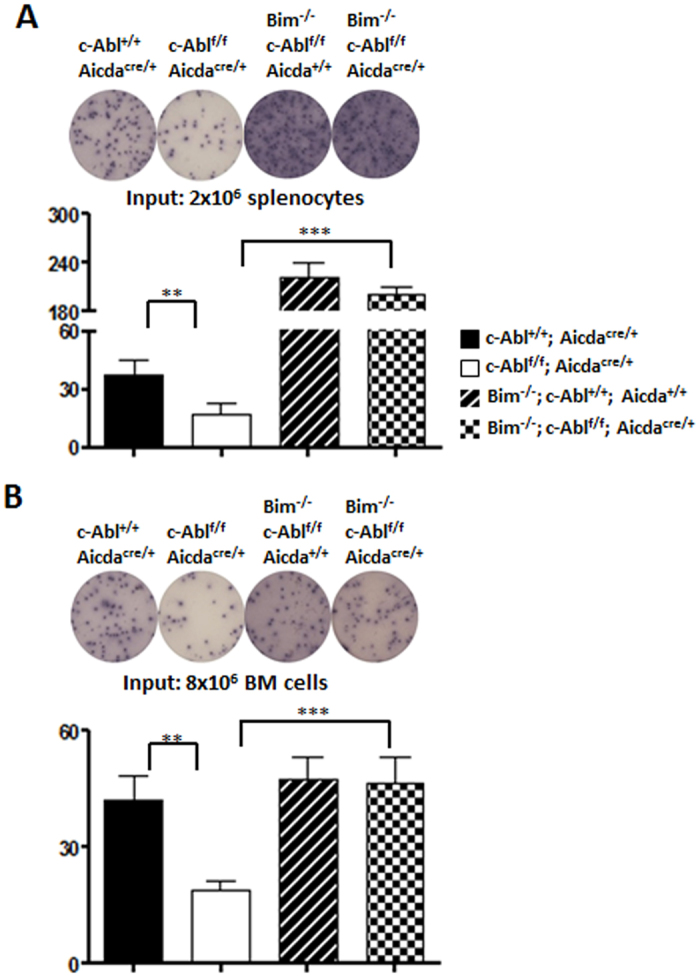
Rectification of PC defects in c-Abl^f/f^ Aicda^cre/+^ mice by introduction of BIM-deficiency. ELISPOT and statistical analyses of NP-specific PC in the spleens (**A**) and bone marrow (**B**) of control, c-Abl^f/f^ Aicda^cre/+^, Bim^−/−^c-Abl^+*/*+^ Aicda^+*/*+^, and Bim^−/−^c-Abl^f/f^Aicda^cre/+^ mice at day 14 post NP_38_-CGG immunization. Data shown are representative of at least three independent experiments.

To further confirm that PC lacking c-Abl have a survival rather than differentiation defect, we introduced Bim-deficiency into c-Abl^f/f^ Aicda^cre/+^ mice. Deletion of the proapoptotic molecule Bim has often been used as tool to determine whether a defective phenotype could be attributable to impaired survival^34^,^35^. We generated c-Abl^f/f^ Aicda^cre/+^ mice lacking Bim and determined if these mice would have increased number of PC. Consistent with the data so far, immunized c-Abl^f/f^ Aicda^cre/+^ mice had reduced PC in their spleens (Fig. 7A) and bone marrow (Fig. 7B) compared with control mice. Interestingly, immunized c-Abl^f/f^ Aicda^cre/+^ Bim^−/−^ mice had significantly increased number of PC in their spleens and bone marrow compared with c-Abl^f/f^ Aicda^cre/+^ mice. Taken together, the data confirmed that PC in immunized c-Abl^f/f^ Aicda^cre/+^ mice exhibit survival defect that results in reduced PC numbers in these mice.

## Discussion

In this study, we generated c-Abl^f/f^Aicda^cre/+^ mice to study the role of c-Abl in B cell terminal differentiation. This mouse strain bypasses the early B cell defects seen in c-Abl total knockout mice and offers us a means to assess the function of c-Abl in plasma cell differentiation. We showed that c-Abl was critically important for the survival IgG1-secreting PCs and this likely involves STAT3 activation. When immunized c-Abl^f/f^Aicda^cre/+^ mice were given a STAT3 activator, Colivelin, the PC defect was largely rectified. The PC survival defect in c-Abl^f/f^Aicda^cre/+^ mice was further confirmed when we deleted the pro-apoptotic BIM and could restore PC numbers in these mice. Thus, c-Abl signalling is critical for PC survival.

Even though our genetic and biochemical data indicated that c-Abl^f/f^ Aicda^cre/+^ mice have an impairment in PC survival, leading to diminished number of IgG1 PCs, we were unable to detect apoptotic PCs in the spleens of immunized mice *ex vivo*. One possible explanation is that even in normal mice PC is a very rare cell population, and further reduction of PCs in the mutant mice would make it technically challenging to detect those apoptotic PCs. We were also not able to detect changes in the expression of survival genes of the Bcl-2 family (Supplementary Fig. 3) in mutant PCs compared to normal PCs, as it is likely that apoptotic PCs could be rapidly and efficiently cleared from the spleen.

Chromosomal translocations of c-Abl such as those that resulted in BCR-Abl underlie the oncogenic transformation of myeloid cells giving rise to myeloid leukemia^28^. Many small chemical inhibitor drugs had been developed to target the Abelson kinases and these include Gleevec, imatinib, desatinib and the newer generations GNF-2 and -5^3^. It will be interesting to determine if dysregulated c-Abl expression or signalling could be implicated in B cell malignancies, in particular, in multiple myeloma (MM) that arises from the oncogenic transformation of PC. Several reports had shown that c-Abl expression was altered in MM compared to normal PC^36^,^37^. Current treatment for MM includes high-dose chemotherapy, use of chemical drugs with broad or undefined specificity such as the proteasome inhibitor bortezomib and stem cell transplantation. Thus, it will be of clinical interest to determine if any of the current Abelson kinases inhibitors could be used, alone or in combinations with other therapies, for the treatment of MM.

Other than MM, knowledge gained in this study could also be applied to the treatment of B cell-mediated autoimmune diseases such as lupus where the presence of autoantibodies causes great harm to the patients. Targeting Abelson kinases activity to eliminate PC in autoimmune patients could potentially alleviate disease burden.

Finally, other than c-Abl itself, components of its signalling pathways could also be potential targets for the development of drugs to treat MM, CML and lupus. Our current study suggested that STAT3 could be one of such targets and STAT3 inhibitors are currently in development or in clinical trials^38^,^39^.

## Materials and Methods

### Mice immunization and treatment

*c-Abl*^*f/f*^ and *Bim*^−/−^ mice were obtained from The Jackson Laboratory. *Aicda*^*Cre*/+^ mice was a kind gift from Nancy Jenkins^40^. *c-Abl*^*f/f*^ mice were bred with *Aicda*^*Cre*/+^ mice to generate *c-Abl*^*f/f*^ *Aicda*^*Cre*/+^ mice, and these mice were further crossed with *Bim*^−/−^ mice to generate *Bim*^−/−^ *c-Abl*^*f/f*^ *Aicda*^*Cre*/+^ mice. Breeding and usage of mice were in compliance with guidelines issued by the National Advisory Committee on Laboratory Animal Research. Experimental protocol involving mice was approved by the Institutional Animal Care and Use Committee (IACUC) of the Biological Resource Center, Agency for Science, Technology (IACUC number 140926). Methods were taken to minimise suffering and improve animal welfare.

To monitor T cell-dependent immune response, sex-matched 6–8 weeks old mice were challenged intra-peritoneally (i.p.) with 100 μg of alum-precipitated NP_38_-CGG (Biosearch Technologies). IgM and IgG1 antibody responses were measured by enzyme-linked immunosorbent assay (ELISA) from sera collected at 7, 14, 21, and 28 days after NP_38_-CGG challenge.

Mice were injected intra-veneously with STAT-3 activator Colivelin (dosage at 1.5 μg/g body weight, Tocris Bioscience, United Kingdom) 5 days after NP_38_-CGG immunization. Mice were given saline buffer for control. Mice were analysed at day 10 post-immunization.

### Flow cytometry

Spleen and bone marrow cells were obtained from mice and single-cell suspensions were extracted. Cells were stained with the following mixtures of fluorochrome-conjugated antibodies: APC-Cy7-B220, APC-CD138, PE-IgD, APC-Cy7-CD19, PE-Cy7-CD38 (BioLegend), PerCP-IgM, FITC-CD21, PE-Fas, FITC-IgG1, PE-CD23 (BD Biosciences). 4′, 6-Diamidino-2-phenyindole (DAPI) (Sigma-Aldrich) was used to exclude dead cells. APC-Cy7-B220, biotin-Dump, FITC-IgG1 and NIP were selected to visualize antigen-specific IgG1 B cells. Biotin-Dump included the following biotinylated antibodies which were then ligated with Streptavidin-PerCP for cell exclusion staining: anti-Th1.2, anti-IgD, anti-DX5, anti-AA4.1, anti-Ter119, anti-NK1.1, anti-CD3, anti-F4/80, anti–Gr-1, anti-CD138, and anti-CD5 mAbs. PE-conjugated NIP-BSA (Biosearch Technologies) was prepared using R-PE labeling kit-SH (Dojindo).

For intracellular staining, cells were first labeled for surface markers, incubated with BD Cytofix/Cytoperm for 30 min, washed with BD Perm/Wash buffer, and then labelled on ice for 30 min with antibodies to fluorophore-conjugated to IgG1, CD138 (BD Biosciences), rabbit IgG (Sigma-Aldrich), c-Abl, phospho-c-Abl (Cell signalling), STAT1, STAT3, STAT5 (eBiosciences), and phalloidin (Thermo Fisher Scientific).

All antibodies were diluted 50–100 times in FACS buffer (PBS buffer containing 5% fetal bovine serum) except for intracellular protein staining, where BD Perm/Wash buffer was used. Data were recorded using LSRII cytometer (BD Biosciences) and further analyzed with FlowJo software (TreeStar). Fluorescence compensation was adjusted to correct for spill-over.

### ELISA and ELISPOT

4-hydroxy-3-nitrophenylacytyl (NP)-specific IgM and IgG1 antibodies were determined by ELISA, and IgG1 PC formation was detected by ELISPOT, following previously published protocols.

For ELISA assay, 384-well flat-bottomed plates (NUNC) were coated at 4 °C overnight with 5 μg/mL NP_14_-BSA (Biosearch Technologies), washed 3 times and blocked for 2 h at RT with 2% BSA in PBS (blocking buffer). Sera sample were serially diluted, added to each well, and subsequently incubated for 2 h at RT. After washing thoroughly for 5 times, biotinylated anti-IgM and anti-IgG1 antibodies (1: 1000 dilution in blocking buffer) were supplemented to each well for 1 h then conjugated with streptavidin-HRP. After adding in 3,3′,5,5′ tetramethyl benzidine thiobarbituric acid substrate, data were collected using tecan microplate reader (Tecan).

To carry out ELISPOT assay, NP_14_-BSA (50 μg/ml, Biosearch Technologies) was used to coat the multiScreen filter plates (Millipore) at 4 °C overnight. Thereafter, 2% BSA in PBS was used to block the plate for 2 h at 37 °C. Single cells were added to each well and incubated for at least 2 h in a humidified incubator containing 5% CO_2_. Subsequently, streptavidin–alkaline phosphatase (AKP)-conjugated anti-IgG1 antibodies were added to the filter plates for 1 h. BCIP/NBT-plus substrate (Mabtech) was used to develop the plate.

### Soluble ligand-binding assay

Soluble ICAM-1/VCAM-1 conjugated to human Fc (20 μg ml^−1^; R&D Systems) were added to the *in vitro*-differentiated PC for 30 min at RT. APC-conjugated anti-human Fc (BioLegend) was used to conjugate ICAM-1/VCAM-1 to reveal the cells that bind the soluble proteins. Data were acquired on a LSRII cytometer (BD Biosciences) and analysed on FlowJo software (TreeStar).

### Confocal microscopy

Spleens sections from immunized mice were embedded in Tissue-Tek and kept at −80 °C till analysis. Cryostat sections that are 8 μm in thickness were dried and fixed in ice-cold acetone for 15 min. Sections were then blocked in blocking buffer containing 5% rat serum plus 2% BSA in PBS for 1 h at RT and FITC-IgD and PE-IgG1 antibodies (diluted 1:50 in blocking buffer) were used for staining (BD Biosciences). Sections were analysed using an Olympus FV1000 microscope (Olympus). Images were taken with Olympus Fluoview Version 2.1 software.

### *In vitro* plasma cell differentiation

Naïve B cells were purified from control and c-Abl^f/f^ Aicda^cre/+^ mice using CD43 magnetic beads (Miltenyi). For PC differentiation *in vitro*, B cells were either stimulated with lipopolysaccharide (LPS) (10 μg/ml) for 3 days or with soluble CD40 ligand (100 ng/ml), IL-4 (10 ng/ml) and IL-5 (5 ng/ml) for 3 days followed by addition of IL-21 (10 ng/ml) and cultured additional 1–4 days before analysis.

### Immunoblot analysis

Lysates prepared from *in vitro* culture were separated by SDS–PAGE and subjected to western blot using standard procedures. Membranes were incubated with horseradish peroxidase-conjugated secondary antibodies (Santa Cruz) and visualized with SuperSignal West Pico/Dura chemiluminescent substrate (Pierce)^25^.

### qRT-PCR analyses

Total RNA was extracted and cDNA was synthesized with the RevertAid H Minus First-Strand cDNA Synthesis Kit (Fermentas). SYBR Green Master Mix (Applied Biosystems) was used for real-time PCR on an ABI Prism7500 instrument (Applied Biosystems). Total mRNA level was normalized against β-actin expression. Primer sequences used were as follows: c-Abl forward, 5′-TCGTTACCTCCAAAGGCTGCTC-3′, c-Abl reverse, 5′-ATGGCGGTGTCTGGCTATTCA-3′; Bcl-2 forward, 5′-GGTCCATCTGACCCTCCGCCG-3′, Bcl-2 reverse, 5′-CCACCACCGTGGCAAAGCGT-3′; Bcl-xL forward, 5′-GATCCAGGAGAACGGCGGCT-3′, Bcl-xL reverse, 5′-TCCTGGCCTTTCCGGCTCTCG-3′; Mcl-1 forward, 5′-CGAACCATTAGCAGAAACTATCAC-3′, Mcl-1 reverse, 5′-AAACCCATCCCAGCCTCTTT-3′; β-actin forward, 5′-AGGCACCAGGGCGTGAT-3′, β-actin reverse, 5′-GCCCACATAGGAATCCTTCTGAC-3′.

### Statistics

Prism (GraphPad) was used to calculate two-tailed Student’s t-test for statistical analyses. P values of less than 0.05 were considered significant.

## Additional Information

**How to cite this article**: Li, Y.-F. *et al*. Tyrosine kinase c-Abl regulates the survival of plasma cells. *Sci. Rep.* **7**, 40133; doi: 10.1038/srep40133 (2017).

**Publisher's note:** Springer Nature remains neutral with regard to jurisdictional claims in published maps and institutional affiliations.

## Supplementary Material

Supplementary Figures
