# Supplementary material for: Tyrosine kinase c-Abl regulates the survival of plasma cells
Source: Sci Rep. 2017 Jan 6;7:40133. doi: 10.1038/srep40133 (PMC5216354; doi:10.1038/srep40133)
Supplement: Supplementary Figures [file srep40133-s1.pdf]

Tyrosine kinase c-Abl regulates the survival of plasma cells

Yan-Feng Li[1]*, Shengli Xu[1,2]*, Huang Yuhan[1], Xijun Ou[1], and Kong-Peng Lam[1, 3]

[1]Immunology Group, Bioprocessing Technology Institute, Agency for Science, Technology and Research, Singapore 138668
[2]Department of Physiology and [3]Department of Microbiology & Immunology, Yong Loo Lin School of Medicine, National University of Singapore, Singapore 117599

Supplementary information

Supplementary Figure 1: Deletion of c-Abl in the germinal center of c-Abl$^{f/f}$ Aicda$^{cre/+}$ mice.
(A) c-Abl$^{+/+}$ Aicda$^{cre/+}$ and c-Abl$^{f/f}$ Aicda$^{cre/+}$ mice were immunized with NP_{38}-CGG and examined 10 days later for intracellular protein levels of c-Abl level in gated GC (Fas^{+}CD38^{-}B220^{+}) B cells in c-Abl$^{+/+}$ Aicda$^{cre/+}$ (red histogram) and c-Abl$^{f/f}$ Aicda$^{cre/+}$ mice (blue histogram). Negative control staining with rabbit IgG antibody (gray histogram) was included. (B) qRT-PCR analysis of gene expression of c-Abl in GC B cells sorted from immunized control and c-Abl$^{f/f}$ Aicda$^{cre/+}$ mice. Data were representative of three independent experiments.

Supplementary Figure 2: Detection of c-Abl in c-Abl$^{f/f}$ Aicda$^{cre/+}$ IgM^{+} plasma cells.
Naive B cells were purified from control and c-Abl$^{f/f}$ Aicda$^{cre/+}$ mice and stimulated with LPS. (A) At day 3 of culture, cells were harvested and analyzed by FACS to identify CD138^{+}IgM^{+}plasma cells at day 6 of culture. (B) qRT-PCR analyses of c-Abl in c-Abl$^{f/f}$ Aicda$^{cre/+}$ IgM^{+} PCs. Untreated naïve B cells purified from c-Abl$^{f/f}$Aicda$^{cre/+}$ mice were used as control. The expression level of c-Abl was normalized to that of β-actin. Data were representative of three independent experiments.

Supplementary Figure 3: Expression levels of anti-apoptotic Bcl-2, Bcl-XL and Mcl-1 mRNAs remained unchanged in plasma cells from control and c-Abl$^{f/f}$Aicda$^{cre/+}$ mice.
qRT-PCR analyses of Bcl-2, Bcl-xL, and Mcl-1 mRNA in sorted PCs from control (red columns) and c-Abl$^{f/f}$ Aicda$^{cre/+}$ (blue columns) mice. Data were representative of at least three independent experiments.

A

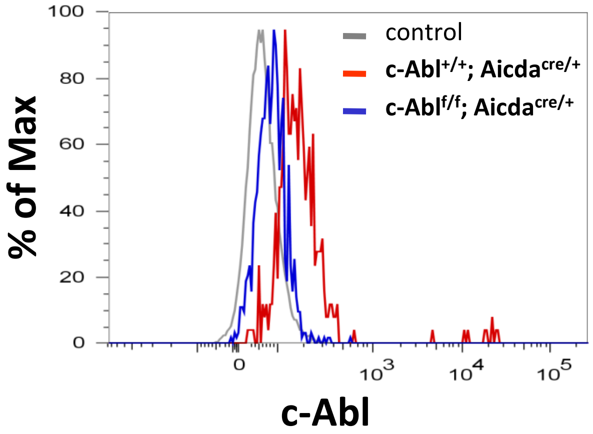

B

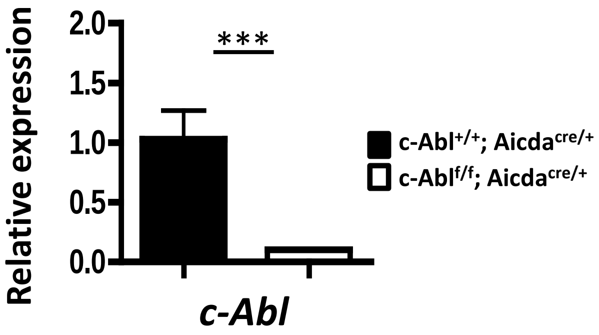

Supplementary Fig. 1

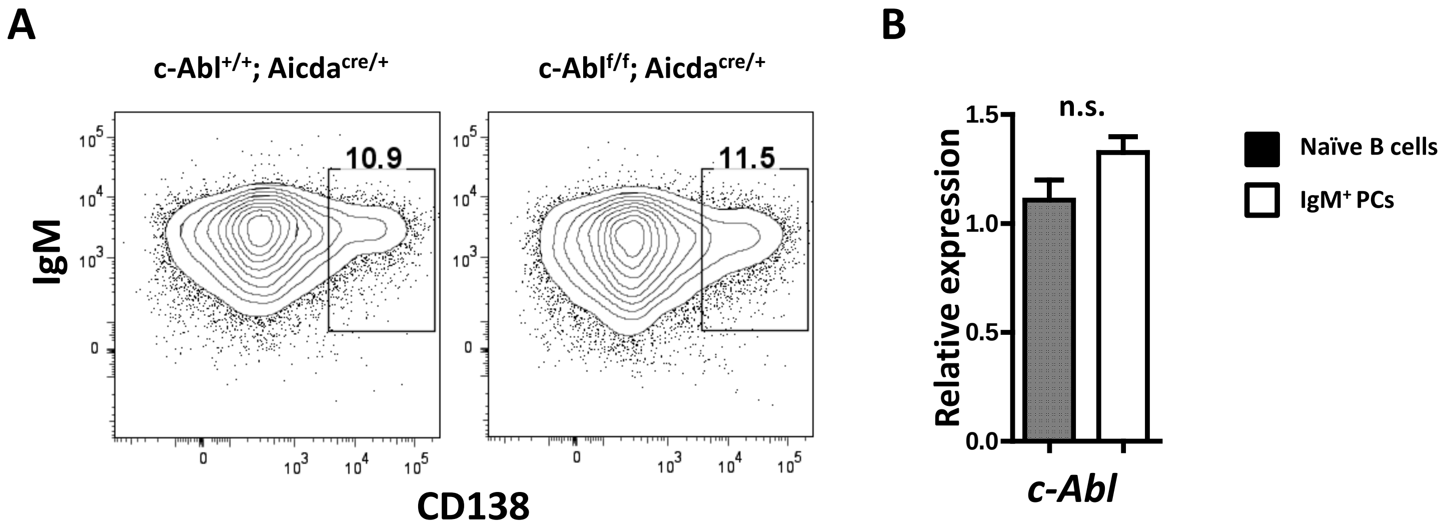

Supplementary Fig. 2

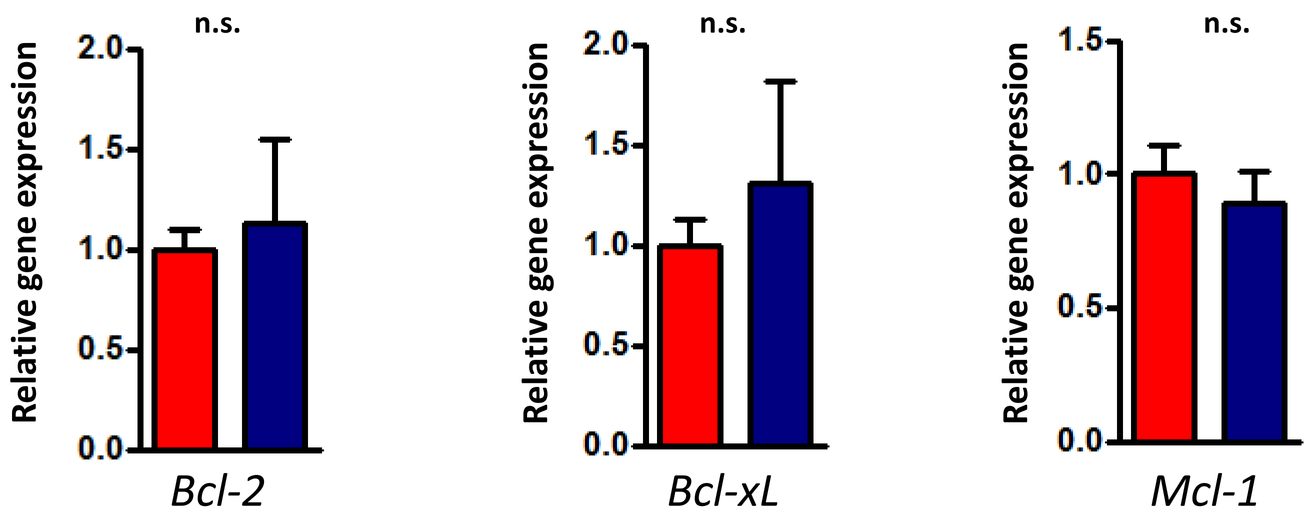

Supplementary Fig. 3
